# Extracts and Gleanings

**Published:** 1875-11

**Authors:** 


### Extracts and Gleanings.

*Menstruation not a Physiological Process.*—Dr. A. F. A. King (*American Journal of Obstetrics,* August, 1875) advances the startling proposition that "menstruation is an abnormal process."

(1) Menstruation is the result of an interference with nature, of a thwarting of her designs, of a violation of her laws, and is preventable by obedience to those laws.

(2) In the great majority of cases, it is not latent, as are other purely physiological processes, but is accompanied by unpleasant symptoms.

(3) To preserve comfort and cleanliness, it requires during its continuance the application of an artificial appendage to the person. This requirement belongs to no natural emunctory.

(4) Menstruation is a hemorrhage; it is attended with the rupture of blood-vessels. Blood-vessels were not made to be ruptured; no hemorrhage is natural.

(5) Although menstruation is desirable and necessary in celibate females to relieve congestion of the uterus, it still ranks second best to reproduction, which prevents abnormal congestion; and it ought no more to be considered physiological on account of its salutary effect, than epistaxis, which relieves congestion of the brain, or bleeding from hemorrhoids, which lessens portal congestion, nor indeed than "vicarious menstruation" from the nose, skin, breast, stomach, lungs, etc., which are equally beneficial in depleting an over-full vascular system.

(6) The menstrual periods in woman are analagous with the periods of œstruation ("heat" or "rut") and ovulation in other animals. In both women and animals these *epochs* are the *periods* naturally designed for coitus and successful impregnation, as evidenced (*a*) by the coincident discharge of ovules, and (*b*) by the well-known greater certainty of conception taking place when coition occurs during the epoch. Now the menstrual discharge, except during the first few ovulatory periods of puberty, prevents coitibn, or if sexual union is admitted without precautionary measures, it may produce gonorrhœa in the male. The menstrual discharge of blood has no analogue in other animals.

(7) Evidence is wanting to prove that menstruation is common in women belonging to the savage races of mankind, who live more strictly in accordance with nature, untrammeled in their reproductive function by the usages of civilization. The Hindoo women, as a rule, do not menstruate; with them menstruation is considered a crime.

(8) History does not furnish unequivocal evidence that menstruation was common in ancient times.

(9) Women have been known to bear large families and enjoy good health without ever menstruating at all. Can it be said that such women are sick? Must it not be admitted rather that they are enjoying a higher grade of health; that their reproductive systems are following a more strictly natural course than belongs to sterile and menstruating females?

(10) Since procreation is natural to women during part of their life, the child-bearing period must have a beginning. If puberty, when the organs are fully developed and prepared to fulfill the procreative office, is not the natural period for reproduction to begin, when else is the beginning of the child-bearing-period?

(11) Organs which are the seat of structural disease, or which are suffering derangement of function, are peculiarly liable to abnormal congestion and inflammation when the individual is exposed to cold. No truly physiological function has this liability entailed upon it. Normal digestion does not prevent the individual from tolerating ordinary exposure, but the dyspeptic with deranged digestion cannot enjoy the same freedom. So exactly normal reproduction does not prevent the female from tolerating ordinary exposure, but she who is the subject of menstruation cannot enjoy the same liberty. The menstrual act here again exhibits the qualities of a pathological process.

I affirm that assuming the female to have attained an approximate or perfect development, she has inherited no tendency to disease, and has been subjected to no such abnormal agencies as would affect injuriously the reproductive organs; under these circumstances there can be no doubt that impregnation during one of the several ovulatory periods that usually precede the establishment of menstruation at the puberic age, is strictly in accordance with nature, and the surest means of maintaining typical perfection, both functional and structural, of the reproductive organs.—*Detroit Medical Review and Pharmacy.*

*Respiratory Percussion.*—In the last number of the *American Journal of the Medical Sciences* Dr. J. M. Da Costa describes a method of physical examination of the chest, by means of which he claims that the diagnosis of pulmonary affections may often be greatly facilitated. By respiratory percussion is meant percussion after a full inspiration or a full expiration, the breath being suspended for the moment while the examination is being made. For the sake of furnishing a standard for comparison between health and disease, a description is given of the sounds produced by respiratory percussion in the normal subject. The general effect of percussion after a full inspiration is to increase the resonance and the volume of sound, and to raise the pitch. Percussion after expiration appears to be of less practical importance, though it occasionally affords valuable information. Its effect is to diminish the resonance and lower the pitch.

The practical application of the respiratory method of percussion would appear to have a very wide range. In all chest affections, where percussion is used, the modification may be employed with advantage. It often serves to clear up a diagnosis where the ordinary physical examination leaves the case doubtful, or where the usual signs tend to mislead. Thus, in bronchitis an abundant accumulation of secretion in the air-passages may obscure the breath-sounds, and even give rise to a certain degree of dullness on percussion But if the suspected region is percussed while the breath is held, after a full inspiration, the percussion-note again becomes clear, and the doubt is removed. On the other hand, should the dullness remain unchanged after inspiration, we may infer that the pulmonary tissue has suffered damage. In acute lobar pneumonia, respiratory percussion may reveal commencing resolution before any crepitation has appeared, and thus become available with respect to prognosis. In certain cases of organic heart disease, where the symptoms lead to a suspicion of tubercular complication, this method of percussion, by causing the local congestion in the lungs to disappear, will greatly assist in the diagnosis. In pleurisy, with effusion, where the fluid is at the lower part of the lung, and some doubt exists as to whether it may not be a case of chronic pneumonia, by means of respiratory percussion the diagnosis may be rendered perfectly certain. If after a forced inspiration the percussion develops a sharply-defined line between the region of dullness below and that of resonance above, it is an effusion; while, if the dullness changes in part, or remains unchanged without being well-defined, we may be sure that the lung is consolidated. Again, in those cases where, in connection with an effusion in the pleura, we get a blowing respiration at the back, with dullness on ordinary percussion, and it is a question whether pneumonia coexists with the pleurisy, a full inspiration expands the lung-tissue, if it be simply compressed or condensed, and the percussion sound becomes clear and resonant.

Obviously in phthisis, too, respiratory percussion may be made of great service in determining the existence of a deposit that is very slight in amount, or in ascertaining the degree of progress of the disease, by magnifying, as it were, the ordinary percussion signs, and by enabling us more accurately to define the limits of the disease. If both lungs are about equally affected, the presence of deposit will be denoted by the fact that respiratory percussion does not give an increased, but rather a diminished resonance, and after a forced expiration the dullness will be markedly increased. In the case of cavities, the effect of a full inspiration upon the percussion sound, is to change the tympanitic, cracked pot, or amphoric notes to simple dullness, together with a higher pitch and a feeling of greater resistance.

In pneumothorax respiratory percussion may be made available, it is claimed, to ascertain whether perforation between the lung and pleural cavity still remains pervious or not. If a full inspiration increases the resonance, the former is probably true; while if the resonance remains unchanged after inspiration, the aperture has closed. This, however, is not stated with complete certainty, and the necessity for further investigation is admitted. In emphysema the vesiculo-tympanitic note elicited by ordinary percussion, is either not at all changed after forced inspiration, or but very slighly so in case the emphysema is not marked.—*Medical Record.*

*Scarless Eradication of Nævi.*—M. Richard Barwell, of Charing-Cross Hospital, thinks that nævus maternus should not be subjected to operation unless some special consideration renders interference necessary. Such consideration may be rapid growth, inducing danger from hemorrhage; or a certain location, over the mastoid process, for instance, where it is liable to become a cirsoid aneurism, or aneurism by anastomosis; or an exposed position on the face, neck, or arm of a female child, where it is extremely unsightly. In the latter case it becomes exceedingly important to choose an operation which shall leave no scar—hence such as shall destroy no skin, not even that discolored by the growth to be eradicated.

The mixed nævus, therefore, requires two forms of treatment, one for the skin, and the other for the subcutaneous portion, and these may be carried on simultaneously. Complete strangulation by ligature is a painful process, frequently causes sloughing of the skin, and, if it does not, then the dead morsel underneath becomes a source of great danger. Several years' experience with many cases leads Mr. Barwell to recommend the following procedure:

"Having carefully made out the limits of the nævus, both as to depth and circumference, a needle, armed with not too fine a wire, is passed through the skin, half round the tumor and out again opposite the place of entrance; the needle is then again introduced, at the same puncture by which it had just emerged, and, passing round the other side of the tumor, makes its final exit at the opening first made. In certain cases, large size or peculiar shape of the tumor may render it necessary to bring out the needle twice, instead of only once. However that may be, the effect is to inclose the base of the tumor in a wire-loop, both ends of which, emerging at the same opening, are under perfect control. These might merely be twisted together until the requisite tightness is attained, but in this practice certain inconveniences arise, which are obviated by another expedient. A vulcanite, oval plate, about three-quarters of an inch long and an eighth of an inch thick, has two holes bored obliquely through its thickness; and on its external surface two little studs project close to where these holes emerge, and where also they are farthest apart. By bringing the end which passes round the right side of the nævus through the left hole, and *vice versa*, the wire is made to cross, while the oblique direction of the holes permits it to run smoothty. The surgeon having thus arranged his appliance, draws upon the wires until the nævus is rather tense, and then twists each end round the nearest stud. A piece of lint, slit so as to bestride the wire, is introduced between the skin and the vulcanite button, and prevents any undue pressure by the edges of the plate."

The wire must be tightened every three or four days, until it has cut through the base of the nævus with its vessels of supply. The child suffers no pain, except while the wire is being tightened, and the small amount of pus that forms escapes through the needle-punctures.

While the subcutaneous portion of the nævus is thus destroyed, the skin will, in many cases, begin to shrivel and lose its morbid color. When this does not occur spontaneously, it is best effected by brushing the colored parts with strong nitric acid, allowing it to remain a few seconds, and then washing it off with an alkaline solution. Care must be taken not to leave on the acid long enough to destroy the skin or produce ulceration. This had best be done while the wire is still encircling the base of the nævus.—*London Lancet, July*, 1875.—*New York Medical Journal.*

*On Croton-Chloral Hydrate.*—In the *Medical Press and Circular*, Dr. J. C. O. Will says:

I may state my decided conviction that, of all hypnotics, croton-chloral has the least troublesome sequelæ.

I make it into a syrup containing two grains of croton-chloral to a drachm of a mixture of glycerine and syrup of orange flowers, colored by adding a very minute quantity of tincture of cochineal. This effectually conceals the taste of the drug, which is certainly to be desired, as it seems to me decidedly unpleasant, and when taken without some flavoring agent it leaves a disagreeable, semi-acid taste in the mouth for a considerable period after swallowing it. This preparation is permanent, a matter of considerable moment, as croton-chloral, though rather freely soluble in warm fluids, is only sparingly so in cold, and when first employing it I was disappointed to find that a mixture which was perfectly clear when first made, soon after became clouded, and threw down a copious deposit of crystals on becoming quite cold. It is, as stated by Walich and Diehl, freely soluble in alcohol, and a strong tincture can thus be prepared; but, unfortunately, on the addition of water, separation soon takes place, the liquid first presenting an oily-like appearance, and soon after depositing crystals. Therefore, if a strong spirituous solution is prescribed, directions must be given that water, in the proportion of at least a drachm to each two grains of the croton-chloral, should be added before the dose is taken, else the changes I have indicated will ensue, and some of the crystals are pretty sure to adhere to the spoon or glass, or to remain in the patient's mouth, an occurrence certainly not disirable, as the taste of pure croton-chloral is far from agreeable.

Case 1.—Mrs. T., æt. 30, suffering from severe facial neuralgia, occurring every night about ten o'clock, was ordered three grains of croton-chloral; half an hour after the pain disappeared, and she slept well, which she had not done for some nights before. On the four following nights the pain recurred at the same hour; three grains were again taken, with similar effect. On the sixth night pain not nearly so severe. On the seventh still less so, after which it did not return. On asking the patient if the mixture made her sleepy, she replied, "No, the pain left me, and then I soon went to sleep." At the time when this statement was made to me I had not seen Liebreich's paper on croton-chloral, but I have since found that it is in accordance with his experience, viz., "that in some cases of tic douloureux theremarkable pheuomenon is exhibited that pain ceases before sleep sets in."

CASE 2.—Mrs. S., æt. 43, a somewhat hysterical female, suffering from supra-orbital neuralgia, appearing every night about eleven o'clock. To take 2½ grains on appearance of pain, to be repeated in two hours if necessary. Soon after the first dose, pain abated considerably; after the second it disappeared entirely, and did not return for some nights; when it did the medicine again acted as on the former occasion.

CASE 3.—Mrs. W., æt. 31, had been for some days attacked by intense pain in her right temple, commencing soon after she arose from bed, and continuing with more or less severity during the greater part of each day. When I was called to her it was more severe than it had ever been before. She was directed to take three grains every second hour till relieved. Six grains sufficed, and when I visited her on the forenoon of the following day she was quite free from pain, and said that soon after the second dose she felt so well that she had been able to serve her customers "just as if nothing had ever been the matter." In this case the truth of Liebreich's statement, already alluded to, was well affirmed.—*Medical and Surgical Reporter.*

*Bromide of Ammonium in Excessive Menses.*—The following suggestions are by Dr. J. K. Black, of Newark, Ohio, in the Cincinnati *Lancet and Observer:*

"The rational mode of controlling certain excesses of the catamenia should be by aiming to remove the conditions upon which these excesses depend. Sometimes this may be from a mere atony or relaxation of the vessels, sequelæ of inflammation or ulceration, or from an abnormal condition of the blood itself; but more frequently is a too frequent or an excessive flow of the menses due, especially in its inception, to a too great excitation of the vasomotor nerves. Whenever this is the case, there is no remedy at all comparable with the bromide of ammonium in controlling the morbid condition. When, without any other obvious causes, the blood being properly organized, the uterine surface not in a state of chronic inflammation or ulceration, there is a too frequent or redundant flow of the menses, either fault will readily yield to the proper administration of this remedy. It appears to act by a direct influence upon the vaso-motor nerves of the generative system, whereby excitement and blood-determination are lowered and lessened, and so tend at once to the establishment of the normal standard.

"I have so often tested the efficacy of this preparation in non-structural catamenial excesses, that I can speak with confidence of its remarkable powers. No more certainly do I anticipate the arrest of an attack of ague by the administration of quinine than do I anticipate the control of the forms of catamenial excess to which I have referred by the proper administration of the bromide of ammonium.

"The other day I visited a young, unmarried lady, who had, for years, been subject to protracted and excessive, though regular catamenial flows. Of late she had displayed serious indications of tubercular disease of the lungs, and, in treating her for this my attention was drawn to the old and exhaustive monthly flows. I am not aware that this excess had ever been mentioned to a previous medical attendant, or that any attempt had ever been made to control it. As the flow usually lasted from a week to ten days, and was quite profuse, it appeared very desirable that its duration and amount should be curtailed, in order to preserve the system, under its new danger, against such a source of exhaustion. Accordingly she was put under the bromide, as follows:

"℞.—Bromid. ammon., . . . . . . ℥j.
Syr. aurantii, aquæ, . . . . . aa. ℥iij.

"M. Sig.—A teaspoonful before tea and at bed-time, commencing ten days before expected period, and continue through it.

"Under this treatment, her mother informed me that she had been a great deal better during the last two periods than had been the case for years.

"In the administration of the remedy, an essential rule is, that its use shall precede the expected period by at least ten days. Its administration only during the crisis will do very little, if any good. The sedative influence of the remedy must precede and accompany the stage of ovarian and uterine vascular engorgement, which itself preceded the flow by several days.

"Some writers have spoken quite favorably of the remedy in dysmenorrhea and menorrhagia, administered in the usual manner; that is, during the crisis only. Having been frequently called to see cases of these disorders during their progress, I have failed to observe any very satisfactory evidence of its controlling-power, while administered only during the emergency. But when administered according to the above directions, it has not only, almost without exception, lessened a regular monthly excess, but it has, in appropriate cases, in quite a number of instances which I can recall to memory, changed a two-week into a four-week crisis."

*Luxation of the Penis.*—The following case is reported in the *Gaz. Méd. de Strasbourg:* A man, fifty-seven years of age, in falling from his wagon, was thrown by his horse against a tree, and the hind-wheel of the wagon passed over his abdomen. On examination, the region of the symphysis, scrotum, and penis, were very much swollen and distorted. It was difficult to find the glans, as it was reduced to a bloody pulp; the penis was flaccid, and seemed to be completely crushed. A sound introduced did not pass beyond the symphysis. On the following day the scrotum was found distended by urine, and perineal section was performed, but the canal was not found; on introducing the finger into the wound a vast cavity was discovered, extending upward to the symphysis and to the left to the inguinal region. From this latter point the urine seemed to proceed. During the days following, micturition was performed about every twelve hours; the urine was clear and inodorous. On the twelfth day an abscess developed at the level of the left anterior iliac spine, and was incised, and from this opening urine escaped in greater quantity than from the perineal wound. A fresh exploration was made on the eighteenth day, and a catheter being introduced into the inguinal wound struck against the root of the penis. The tissues were incised on a director, and at the bottom of the wound the penis was discovered intact and strongly adherent to the cellulo-fatty tissue covering the abdominal muscles. The glans had been separated from the preputial fold and had assumed a direction toward the spine of the ilium, the entire penis having left its sheath. In other respects the canal had not sustained any alternation. The patient refusing to submit to any autoplastic operation for the better isolation of the penis, treatment was confined to removing the skin and fat which surrounded it within a certain compass. The patient was again seen one year afterward; then the penis had become adherent to the skin of the abdomen almost up to the glans; the latter was mobile, and micturition could be performed in every position. Erections took place from time to time, and were not painful. This case and the one of Nélaton, in which the penis was dislocated into the scrotum, are the only two cases of this kind on record.—*Reveue de la Presse Médicale.*—E. F. *in New York Medical Journal.*

*Influence of Anæsthetics upon the Sexual Impressions of Females.* A physician, called as an expert before a United States tribunal, made the following declaration: "A woman under the influence of anæsthesia is more liable to conception than when sexual intercourse has happened by force, and I concur in the opinion of Dr. Beck, expressed in his treatise on medical jurisprudence, that women may conceive during anæsthesia. The relaxation it produces facilitates conception."

This point seems to me established; but I desire to add an observation which I have made in my practice, and one that deeply concerns physicians to know. It is well known to-day that occasionally, under the influence of ether or chloroform, an excitation of the sexual organs is produced, and a feeling is excited in the mind by this sensation which may make a woman believe that she has been subjected to violence.

The first case of this nature which I witnessed myself occurred during a delivery. The woman, placed under chloroform, experienced sexual sensations so vivid that she accused me of having violated her, and called on her husband for protection. But he had been with her all the time, as well as a dozen women who had never quitted the chamber. In a second case I was administering chloroform to a woman to have a tooth extracted, but the physiognomy of the patient showed an expression of venereal excitement so pronounced that I hastened to call in her parents. On awakening she seemed astonished to see herself surrounded by her family and clearly exhibited what her impressions had been.

On another occasion a lady of a certain age entered my office in a state of high excitement, and related that she had gone to her surgeon to have a trivial operation performed, to relieve the pain of which she had taken chloroform, and the surgeon had abused her while under its influence. I was persuaded that she had deceived herself, and, on examining all the circumstances, clearly proved to her that she had been subject to a delusion.

The moral is that physicians should never administer ether or chloroform except in the presence of witnesses.—*Reveue Medicale, August* 18, 1874.

*Common Colds* (*The Practitioner*).—Dr. J. Milner Fothergill briefly considers the causes and the treatment of common colds. Whenever and wherever they are met, they are the consequence of a chill, either to the general surface or to a portion of it. Ordinarily the body-temperature is maintained by the equilibrium existing between the internal heat-producing area and the external heat-losing area, or the surface. When excessive heat-loss is not met by increased heat-production, a chill or lowering of the body-temperature is the consequence, or if heat-production has been great, the cutaneous vessels are dilated, and if the surface be suddenly exposed to cold, these dilated vessels are apt to be paralyzed instead of incited to contraction, and then heat is rapidly lost from the mass of warm blood in the cutaneous vessels. The catching cold or the escape from doing so depends upon the state of the vessels of the surface and their capacity to contract or the opposite. Consequently, we can see that catching cold or escaping from it under apparently identical circumstances depends upon a condition far removed from either vision or sensation. Where heat-loss is met by heat-production at the time, no unpleasant consequences result; but where the heat-regulating processes are delayed, the loss of heat and fall of temperature at the time are followed by an excessive heat-production, constituting a pyretic condition which in its simplest form is a cold.

Obviously, the indications for treatment are to restore the balance between the heat-producing and heat-losing areas; and in order to do so we resort to such measures as shall increase the amount of blood in the outer area, and so diminish the amount in the internal area; that is, to increase heat-loss and lessen heat-production. We consequently use hot fluids, nauseant diaphoretics, warm beds, opium, and quinine—the latter usually after the action of the skin has been re-established.—*Medical Times.*

*Mercurial Baths in Syphilis* (*The Lancet*, August 21, 1875).—Mr. Henry Lee describes as follows the plan of administering the mercurial vapor-bath, which he extols highly as comprising, in many respects, the greatest therapeutic advantage with the least possibility of unpleasant effect.

A lamp, in which the methylated spirits of wine is burned, is put into a case, made principally of wire gauze, on the principle of the Davy safety-lamp. The top of the case is fitted with a central, movable, small circular plate, surrounded by a trough, which should contain one ounce of water only. The water should be boiling when first put in, or should be allowed to remain over the lighted lamp until it begins to boil. Thirty grains of re-sublimed calomel are then spread out on the central small circular plate. This should be quite dry. The patient then sits, without his clothes, on a small stool or chair, and the lamp is placed between his legs. A cloak made of moleskin or of some thick material is then made to cover the whole apparatus, and is tied round the patient's neck. It is important that the cloak should go quite down to the ground all the way around. As the water boils, a certain quantity of steam is enclosed within the cloak, and a little later the vapor of the calomel as it rises passes through the steam and becomes mixed with it. The water first disappears, and the calomel is sublimed in from ten to fifteen minutes. The patient then gets into bed with the cloak on, so as to make it his night-dress. In this way the calomel is necessarily kept on the surface of the skin. The cloak used is furnished with a cane-hoop, so as to be kept away from the skin during the action of the bath, and this hoop may be removed as soon as the bath is over, and replaced again before the bath is used the next night. The cloak has a slit in front, which the patient is generally directed to open for about an inch, so as to allow some of the vapor to escape. This rises in front of his mouth and nose, and he is directed to inhale it for a minute at the expiration of each five minutes during the continuance of the bath, so as to breathe the vapor for about three minutes altogether. The patient during this time keeps his head up, so that the moistened calomel vapor passes for about six inches through the common air before it is inhaled. This inhalation is not always necessary, but it furnishes a means of regulating with the greatest nicety the action of the mercury, as indicated by its effects upon the gums. He has never found mercury administered in this way produce salivation where patients had not also taken it in some other form. The action is upon the surface of the body, and the internal parts are comparatively unaffected. No diarrhœa is produced except from some accidental cause. The stomach and intestines are not irritated, and are free from the use of food or medicine. The perspiration produced amounts only to a slight moisture on the skin, and when this is the case the patient very rarely experiences any debilitating effects from the continued use of the bath. During this treatment Dr. L. generally recommends patients to abstain from taking vegetable acids; and for this purpose, as a rule, they are told not to eat raw vegetables or raw fruits, such as salads, cucumbers, celery, apples, pears and oranges. As the object is to have the calomel in contact with the skin, the patient washes only as much as may be necessary.

*Diminution of the Uterus after Delivery.*—In an elaborate article on this subject, communicated to the Obstetrical Society of Edinburgh (*Edinburgh Medical Journal*, May, 1875) Dr. Ar. Serdukoff arrives at the following conclusions:

1. Involution of the uterus goes on more rapidly during the first few days of the puerperal period than it subsequently does.

2. Involution of the uterus of healthy women goes on well and with regularity.

3. Involution, where the uterus is the subject of disease, such as metritis, endometritis, or parametritis, goes on more slowly, and this varies with the amount of disease.

4. The permanent contraction which takes place during the first few hours after delivery is a common occurrence. When it passes off an increase in size begins to take place.

5. In women delivered at the full time, involution goes on more quickly and regularly than in those prematurely confined.

6. Length of labor retards involution.

7. In primiparæ involution of the uterus goes on very regularly, but more slowly than in young multiparæ. In aged multiparæ involution does not go on so well.

8. In women who suckle their children, involution during the first four days does not go on so quickly as in those who do not nurse. But subsequently, the involution is quicker, though less regular.

9. After-pains are not necessary for a favorable involution; in fact, we are as well without them.

10. In order to determine the involution of the uterus, you should only measure its breadth.

11. Involution of the uterus goes on proportionately in length as well as breadth.

12. Super-involution and sub-involution occur as distinct, uncomplicated pathological conditions.—*Monthly Abstract Medical Science.*

*Cause and Prevention of Typhoid Fever in Schools.*—Toward the close of the last year, a serious epidemic of typhoid fever broke out in a well-known boarding-school for young ladies, at Burlington, New Jersey. For the purpose of ascertaining the causes which led to the outbreak, the trustees of the institution engaged the services of Dr. J. L. Leconte, as a sanitary expert, who now presents the results of his investigations in a communication to the Philadelphia *Medical Times,* (May 29, 1875.) An examination of the premises of the school elicted the following facts: Two large cisterns were situated near the school, from which the water-supply was obtained. In building the cisterns, it was necessary to cut holes in them in laying the floors, in order to relieve the pressure from the spring-water, the floors being below the level of the subterraneous drainage. These holes were afterward plugged, and the water-supply was derived entirely from the river. A year later, for some reason, the plugs were removed. Some three years ago, privy-vaults were built, one of which was incautiously placed within a dozen feet or so of one of the cisterns. Leconte infers that matters gradually oozing from the vault had little by little contaminated the surrounding soil, until they finally gained access to the cisterns through the holes from which the plugs had been removed, and so polluted the water. As evidence that the polluted cistern-water was the sole cause of the epidemic, it is mentioned that as soon as, at the suggestion of the physician of the school, water only was used that was drawn directly from the river, not another case of the disease occurred. Several other significant facts are mentioned also. For instance, it was learned that no cases of typhoid had occurred among the servants, and on inquiry it was ascertained that they had not been in the habit of drinking the cistern-water, excepting in tea and coffee; that is, after it had been boiled. Moreover, the majority of the girls who had been attacked had drank water alone—were not tea and coffee-drinkers.—*Medical Record.*

*Successful Ovariotomy Operation Performed Under Numerous Difficulties.*—Dr. S. G. Stevens, traveling in Central America, without any expectation of being called on to perform surgical operations, had with him only an ordinary pocket-case; he was requested to remove an ovarian tumor. He made a trocar from a piece of bamboo, to which he attached an elastic tube, and went in search of assistance. With difficulty he obtained the services of a Catholic minister, two Indians, and a tradesman; but, at the moment of commencing the operation, he found that he had no chloroform. Chloroform was not as easily made as a bamboo trocar. Dr. Stevens then left with his four assistants, accompanied by the patient, to search for the precious anæsthetic. It required a journey of five days in order to find it. He then at once began to get ready for the operation. He commenced himself to give the patient chloroform, and afterwards confided its administration to the missionary. The latter, owing to his desire to see the operation, performed his allotted duty imperfectly, and Dr. S. proceeded with the operation, emptied and removed the tumor, which was an enormous polycyst. He tied tightly the pedicle with a thread of hair, and left it in the abdominal cavity; afterwards he cleaned the latter with wadding and closed the wound with iron wire. The patient rapidly recovered.—*La Tribune Medicale.*

*Hot Baths.*—Professor Lasegue lays down (*Medical Press and Circular*) the following rules: No hot bath ought to exceed twenty or thirty minutes in duration. The initial temperature ought always to be lower than the final temperature. The increase of temperature ought always to be gradual. The maximum temperature is usually 103°, but 108° can easily be tolerated if the patient does not remain in this temperature longer than eight or ten minutes, and if the unpleasant sensation produced by the vapor on the part of the body which is not immersed is avoided. On leaving the bath the patient goes to bed, and soon loses the sensation of unusual heat. Cold douches, which are so agreeable after hot-air bat[illegible] are not well borne after hot-water baths. Lasegue has found a [illegible]olonged course of hot baths very useful in chronic rheumatic arthritis. Under their influence the movements of the articulations have become less difficult and painful. A similar mode of treatment has been found useful in chronic abdominal complaints, such as protracted diarrhœa, and even in obstinate chronic bronchitis. *The American Practitioner.*

*Ergot—Modes by which it Destroys Uterine Fibroids.*—Dr. W. H. Byford (*Medical Examiner*, July 1, 1875) concludes an elaborate paper upon the treatment of uterine fibroids with ergot, by the following statements:

1. It is gradually disintegrated and absorbed. In this way it disappears without any violent or disagreeable symptoms.

2. Its nutrition is so interrupted as to produce a rapid destruction of its vitality, and hence decomposition within the capsule, and a semi-putrid mass expelled. This process is accompanied with evidences of inflammation of the uterus and toxæmia, more or less grave, according to the size of the tumor, the length of time between the commencement of decomposition and the expulsion of the tumor, and the vital resistance of the patient.

3. The tumor, in nearly its original condition, is totally or partially expelled from the cavity of the uterus, attended with varying degress of inversion of the organ. In this condition it becomes amenable to surgical process for completing its removal.—*Indiana Journal of Medicine.*

*Secondary Syphilis.*—℞—Hydrarg. bichlor. grs. ij; Pot. Iodidi. grs. xl; Syr. Surzæ co. ℥j; aquæ *ad* ℥viij. Sig.—A tablespoonful three times a day.—*Canada Lancet.*

*Local Treatment of Cancer.*—Dr. J. E. Nichols recommends, in the Chicago *Medical Journal,* the more frequent use of local applications in cancer. We quote one case to show his treatment:

Case 1.—Mrs. J. B., aged fifty years, sent for me, December 10, 1873; has been scrofulous and an invalid several years; no cancer in her family. For several months a small, hard, painful tumor has been coming on the gums of incisor teeth and roof of the mouth, obviously caused by wearing false teeth on an illy-fitting rubber-plate. Some five weeks before, a little-pill fellow had bunglingly endeavored to extirpate it with a pair of scissors [illegible]d table-fork. It had grown very rapidly from the time of that mutilation, and was now as large as a walnut. Diagnosed it to be an epithelial cancer. Treatment:

℞.—Chloride of zinc.
Pulv. bloodroot, aa. q. s.

Rub together in open air until it forms a stiff mass or paste. Put enough on cotton-wool to cover the entire surface of the cancer; wedge more cotton-wool between it and the upper lip; tell her to avoid allowing the saliva to come in contact with it as much as possible, and instruct her to remove it and have the paste wiped entirely out of the mouth when she has had it there long enough to become tired.

December 11th. Patient has left the paste in position about two hours, and on examination I found a thin, white eschar over the entire surface of the tumor, and detached and loose in places. Pocketed some paste under the loose parts of eschar, and applied more on the surface as before. I returned in about two hours and removed it myself.

I repeated this process for ten days, each day removing detached parts of the eschar, and applying fresh paste, both by pocketing it under loose parts of the eschar and on cotton-wool. At the end of this time, the wound presented that freedom from cancerous particles that one soon learns to recognize in using this paste. The applications were quite painful at times, but not dreaded as would have been the knife.—*Medical and Surgical Reporter.*

Add a little borax to your salicylic acid, if you wish to dissolve much of it in water.

*New Material for Fixed Dressings.*—Dr. R. J. Lewis (*American Practice*) recommends glue and oxide of zinc as the one dressing which fulfills all requirements; being cleanly in its application, drying with sufficient rapidity, removable without difficulty, exceedingly light, and withal very cheap. The material is ordinary glue, with which oxide of zinc has been incorporated at the time of using it, in order to cause it to harden rapidly. Several pieces of flannel—old blankets or worn-out under-clothing answering the purpose admirably—are selected and cut to the requisite size. One of these is laid around the limb, and the two edges are tightly stitched together along the anterior surface, allowing the edge to project above the seam; then the melted glue with oxide of zinc is painted upon this with a brush. The dressing may be strengthened by an additional layer of flannel, or blanket, saturated with the glue and oxide of zinc, and made to adhere to the underlying layer. A third or even a fourth layer may be thus applied, if it is deemed necessary, and the limb supported until the dressing dries, which requires from four to eight hours. The stitches of the seam on the front of the limb having been cut with scissors, the edges of this elastic case are sprung apart, and the dressing removed. The edges are then trimmed smooth and a number of eyelets inserted, in order that the case may be laced like a shoe, and the degree of pressure regulated. The fixed fracture-apparatus is exceedingly light, is made from material almost everywhere obtainable, and is much cheaper than the silicate dressing. Another advantage is its elasticity, which permits its removal without endangering the splint, for it can be pulled apart, and immediately sprung into place around the limb to which it has been moulded. By a little care and dexterity in stitching on the layers of flannel the surgeon can readily shape the dressing so that both the leg and foot are completely encased.

*A New Adhesive Plaster.*—Kauvin recommends a mixture of twenty parts of mucilage of gum arabic and one part glycerine, spread three or four times upon linen, at sufficient intervals to allow it to thoroughly dry. This plaster is glossy, pliable, does not deteriorate with keeping, and is considerably cheaper than English plaster, or diachylon plaster.—*Exchange.*

*Subcutaneous Injection of Strychnia in Incontinence of Urine.*—Dr. Kelp, director of the lunatic asylum at Wehnen, near Oldenburg, describes the results of subcutaneous injection of strychnia in two cases (Deuts. Arc. fur Klin. Med.) One case was that of a girl aged sixteen, who was admitted with melancholia stupida and was discharged cured at the end of about a year and a half. She was small and feeble and had been badly cared for at home. From childhood she had suffered from nocturnal incontinence of urine. Small doses of strychnia, given internally, produced no effect. But, after the injection of one-sixteenth of a grain of nitrite strychnia dissolved in water (one grain in two drachms) in the sacral region, the enuresis ceased, and did not return for some days. The injections were repeated from December 17, 1867, to April 3, 1868, whenever the enuresis returned; it appeared at gradually increasing intervals, and at last ceased. The dose of strychnia was increased to one-sixth of a grain. During the whole time not more than four grains were used. For a year after her discharge she resided near the hospital, and was occasionally troubled with incontinence of urine, which was removed by repetition of the remedy. She has since married and has not come under Dr. Kelp's notice, from which he concludes that the enuresis has not returned. The second patient was an imbecile girl, aged twenty, who had suffered from enuresis from childhood. The treatment was the same as in the former case, and the result was as good; but Dr. Kelp does not know whether the result is permanent, as he has not heard of the patient since she left the asylum. The treatment was continued for about two years, during which about ten grains of strychnia, in all, were used. The intervals between the attacks of enuresis varied from some weeks to a few days. The usual dose was one-seventh or one-eighth of a grain; but, even when this was used daily, no general action on the system was observed.—*British Medical Journal.*

*For Ingrowing Toenail.*—Dr. D. S. Fiske, of Brookfield, Massachusetts, writes: "Remove a V-shaped piece of the fleshy part of the toe, so as to bring the nail over the outer part of the toe, making a flap of this outer part, and it will make a narrow toe and remove the difficulty better than any operation I have ever tried." *New York Medical Record.*

*Acid Tannate of Iron in Diphtheria.*—Sometime in August last my attention was directed to an article in the *Physician and Pharmacist*, by Dr. J. A. Hopkins, of Milton, Delaware, regarding the local use of the acid tannate of iron in diphtheria. Though the disease has not been very prevalent throughout my immediate neighborhood since my experience with the substance in some six cases has been so favorable as to induce me to second the doctor in calling the attention of the profession to it as a valuable therapeutic agent. In one case, especially, an effect was produced which may be of interest. The patient being a docile, quiet girl, whose tonsils were completely covered with the diphtheria deposit, I introduced the point of a bistoury, and gave a little slit, by means of which I was enabled to detach and hold up a portion of pseudo-membrane, about a line in thickness, and having the appearance of "dirty wash-leather." On applying the acid tannate, a rapid apparent coagulation took place, the membrane shrinking from the incision towards the periphery. Nor was it afterward reproduced to any great extent. In other cases no such striking result was obtained, but the local disease would appear to be modified, the influence of the agent being attested by the at first blackened and afterward brownish color of the deposit. Exfoliation generally began in the course of a few hours, in the form of hardened patches coughed up and expectorated occasionally. The method of application which I found best, was first to apply thoroughly the "tr. ferri chl.," followed immediately by a strong solution of tannic acid; thus allowing the chemical reaction to take place on the diseased portion. An application once in the twenty-four hours is, I think, sufficient, due attention being of course paid to tonic and stimulating remedies, with disinfectant solutions and nourishment.—H. B. WITHERHORNE *in the Medical Record.*

*Traumatic Inflammation of the Liver.*--Alex. Uwersky found that the liver cells take no active part in case of inflammation, but become destroyed by retrograde metamorphosis. The pus cells which may be observed are emigrated colorless blood corpuscles. A new formation of connective tissue as a product of inflammation is probably caused by colorless blood corpuscles.—*Virchow's Archives, April,* 1875.

*Lingering Labor from Rigidity of the Soft Structures.*—I wish to call the attention of the profession to the use of two remedies in rigidity of the parturient canal, namely, lobelia-seed and gelsemium. My experience with these two remedies has been quite flattering. It will not, however, do to use them in all cases indiscriminately. I have found them useful in two very opposite states. If the os is full, thick, hard, unyielding, giving a moist, cool, clammy sensation to the finger, and the same pertains to the vagina and perineum, with a limited secretion of mucus, use lobelia. The skin will also present a full, cool, clammy feeling, while the pulse will be slow, full, and oppressed. When I meet a lingering case of labor, where the bony pelvis is competent to pass the child, presenting the above conditions, I use the tr. lobelia-seed in from three to ten-drop doses every one-half or one hour, until the tissues relax. Emesis is not desirable, and, when threatened, lessen the dose Given the above conditions, and I have yet to see the case of rigid parturient structures which will not yield to tr. lobelia-seed.

When the os, vagina, and perineum are thin, hard, dry, and contracted, giving to the finger a sensation of dryness and pungent heat, use fl. ext. gelseminum, green root. The woman will be restless and irritable; skin dry and hot; pulse sharp, vibrating, and accelerated. I give from ten to twenty drops at once, and follow it with five-drop doses every one-half or one hour. It is not best to employ too large doses, or carry them to the extent of producing toxic symptoms; small doses frequently repeated are better than large ones at greater intervals. My experience is that these two drugs never fail when the above-mentioned symptoms are present. C. L. GREGORY, M. D., *in Cincinnati Medical News.*

*Bone-Dust as Food.*—It is mentioned in the report of the school for rickety children, recently established at Milan, that great benefit has been derived from the use of powdered bone administered in milk.

DR. E. H. TRENHOLME reports (*Canada Medical Record*) a case of traumatic tetanus successfully treated with chloral and bromide of potassium.

*Ether or Chloroform?*—In the session of the Medical Society of Florence, Professor Schiff stated that in ether-narcosis a gradually progressing suspension, first of the respiration and then of the circulation, sets in; while in chloroform-narcosis this succession is not constant, but that the circulation often stops while the breathing continues. In ether-narcosis, therefore, the physician is responsible for the death of the anæsthetized subject, but not in chloroform-narcosis.—*Centralblatt fuer Chirurgie.*

*Gargle for Sore Throat.*—Dr. F. A. Burrall, of New York, says that for two years he has had experience in practice in the use of the following gargle, which is especially serviceable when used early in sore throat, when it seems, sometimes, to abort the attack:

℞.—Bromo chloralum glycerin, . . aa p. æq.
Tr. cocci cacti, . . . . . . qs. M.

Two teaspoonsful in a goblet of water used as a gargle every half hour.

*Ointment for Sycosis.*—Dr. S. Smith, of New London, Connecticut, sends us the following formula for an ointment, which he has used for several years, with unvarying success, in the cure of this intractable affection:

℞.—Acid tannic, . . . . . . gr. xv.
Sulphur, . . . . . . gr. xij.
Aquæ rosæ, . . . . . . qtt. xviij.
Adepis, . . . . . . . ʒ ijss. M.

*Gelseminum in Odontalgia and Facial Neuralgia.*—Dr. J. Sawyer, of London, and Dr. E. Mackey, of Birmingham, extol (*British Medical Journal*) the efficacy of tincture of gelseminum, made from two ounces of coarsely powdered gelseminum root, macerated in a pint of rectified spirit, for the relief of odontalgia and facial neuralgia. The dose is from five to twenty drops every six hours. Dr. Sawyer says that out of about twenty cases the use of the remedy has not failed to give decided and lasting relief in more than three or four.—*American Journal of Medical Sciences.*

SACCHARATED Dover's powder is made by substituting milk-sugar for potassium sulphate, in the regular formula. It does not "coagulate" with sugar-of-lead, and is in that respect superior to the time-honored formula.—*Ex.*

*The Danger of Cantharides.*—We see it stated in the dailies that two young men of Warsaw, Indiana, gave a young woman a dose of wine and cantharides, for an infamous purpose, and caused her death. They acknowledged the act, and are in jail. Physicians should take proper occasions to disabuse the public mind of the belief that cantharides is an aphrodisiac, and also to warn of its dangerous character. Vile publications have disseminated a belief that it is a true venereal excitant, which it is not in any sense. *Medical and Surgical Reporter.*

*Medicated Milk.*—At Nice, in France, there has been recently established a dairy under the name of the "Medical Dairy," and it is stated that it has introduced a new system of taking medicine homœopathically, so as still further to carry out the minimization of infinitesimal doses. Goats are kept, and when any patient requires a particular medicine, it is first administered to the animal, and some of the milk is afterward taken by the invalid. Some goats are kept for particular medicines, so that there is the belladonna goat, the arnica goat, the camphor goat, etc.—*Ex.*

*Catarrhal Affections.*—The following is a favorite prescription for catarrhal pneumonia, bronchitis, etc.:

| | | |
|---|---|---|
| ℞.—Ammon. muriat., . . . | ʒ ij. and ℈ ij. | |
| Potass. chlorat, aa., . . . | ʒ ij. | |
| Aq. aurant. flor., . . . | ʒ ij. | |
| Syr. simplic., . . . . | ℥ ij. | |
| Aqua, ad., . . . . | ℥ iv. | M. |

S. Two teaspoonsful t.i.d.

*Ergot in the Treatment of Insanity* (*The Medical Record, June* 26, 1875).—Dr. Edward C. Mann reports several cases of maniacal disease in which the free exhibition of ergot produced either a decided amelioration or a positive cure. He believes with Dr. Brown that it is eminently useful in recurrent mania, chronic mania with lucid intervals, and epileptic mania, and that its good effect is due to its power of re-establishing the equilibrium of the intra-cranial circulation which has been temporarily disturbed.—*Medical Times.*

*Solubility of Salicylic Acid in Glycerine.*—One part of salicylic acid dissolves completely in fifty parts of cautiously-heated glycerine, the solution remaining clear after cooling, and may be diluted without separating the acid. A solution made with one part of salicylic acid, 20 to 30 parts of glycerine, and 300 to 500 parts of hot water, has been used for sometime in the surgical ward of the Bremen hospital.—*Phar. Zeitung and American Journal of Pharmacy.*

*Treatment of Ozœna by Injections of Chloral* (*Bull. Gen. de Therap.*, *July* 30, 1875).—M. Créquy reports the cure of an obstinate case of ozæna by the injection of a solution of chloral. He simply made a siphon of an india-rubber tube by putting one end to the injection and the other well up into the nasal cavity. The disease had resisted injections of tannin, phenol, corrosive sublimate, etc., but was cured in a short time by this method.—*Medical Times.*

*Solution of Morphia for Hypodermic Injection.*—Dissolve ten grains of hydrochlorate of morphia in two drachms of distilled water by the aid of heat, without any acid, spirit, or glycerin. Two minims cf this solution, that is, one-sixth of a grain, should be the commencing dose. It becomes solid at ordinary temperatures, and when wanted for use must be heated. The advantage is, that however long it is kept, the solution never spoils.—*Canada Lancet*, April, 1875.

*Syrup of Acacia.*—C. B. Mann recommends the following formula:

| | |
|---|---|
| ℞.—Acacia, . . . . . . | ℥ ij. |
| Glycerin, . . . . . . | f.℥ j. |
| Water, . . . . . . | f.℥ vij. |
| Sugar, . . . . . . | ℥ xiij. |

Mix glycerin and water; dissolve gum; add sugar; boil and strain.

*Diagnosis of Ovarian Disease.*—Mr. Spencer Wells called attention, at a recent meeting of the London Pathological Society, to the fact that while extra-ovarian cysts are often radically cured by a single tapping, the cyst contracting and never refilling, true ovarian single cysts are almost certain to fill again. The contents of parovarian cysts consisted of little more than pure water.—*New York Medical Journal.*

*Ascarides Treated by Enemata of Cod-Liver Oil.*—Five tablespoonsful of the pure oil were prescribed by Szerleki, as an enema, to be used twice daily. The effect of this treatment was primarily to bring away many living worms, and, after being continued for some days, to relieve the patient wholly of the itching and soreness in the perineum, and of the resulting fever, by which he had been very mucn reduced.—*Medical Record*, January, 1875.

*Remedy for Chronic Hoarseness.*—The *American Practitioner* states, on the authority of an eminent physician of Philadelphia, that in chronic hoarseness, arising from thickening of the vocal chords and adjacent membrane, the ammoniated tincture of guaiacum is often a very efficacious remedy. It may be appropriately mixed with equal parts of the syrup of senega, and a teaspoonful of the mixture given two or three times a day.

*Retained Placenta.*—Dr. Linéard, of Caen (*Gazette des Hopitaux*) recommends in retained placenta, and for the prevention of after-pains and uterine hemorrhage, the injection of the umbilical cord with cold water by means of a syringe.—*New York Medical Journal.*

*To Promote Sound and Tranquil Sleep.*—Dr. Ritman recommends rubbing the abdomen with the hand for eight to fifteen minutes. A glass of cold water before retiring is also recommended for the same purpose.—*Baltimore Physician and Surgeon.*

*Asthma.*—The iodide of potassa is frequently an efficacious remedy in asthma, given in two and four-grain doses every four hours. Its value is much increased by combining it with ammonia arb., five grains.—*The Archives.*

